# Evolution of complexity through regulatory variation at a single gene

**DOI:** 10.1073/pnas.2424050122

**Published:** 2025-01-21

**Authors:** Mafalda S. Ferreira

**Affiliations:** ^a^Department of Zoology, Science for Life Laboratory, Stockholm University, Stockholm SE-10691, Sweden

How does biodiversity originate? What is its function? How does it change over time by interacting with the environment or other diversity? The answers may allow us to predict the direction in which a trait will evolve in response to environmental change. The quest for this predictive power is old (1) and is still underway (2, 3). Part of the quest involves determining how traits are built, from physiological and hormonal changes, to tissues and cell types, to proteins, genes, and mutations (4). All of these components act in coordination to construct a functional organism, and changes to one or various of these components create diversity. Selection acts on this diversity, which can fundamentally alter the evolutionary trajectory of populations and species. Therefore, it is important to know how traits are built—mechanistically—to be able to predict evolutionary outcomes.

In the last few decades, the field of genomics has allowed researchers powerful insight into linking trait diversity to regions of the genome, or even particular genes and mutations, in model and nonmodel organisms (5). While progress has been made in understanding “what” drives trait variation, less is known about “how” genetic regions, through their functional roles, generate this variation. In PNAS, Verta et al. (6) use state-of-the art genomics to reveal how a single gene determines the life history strategy of salmon through its multifaceted control over molecular pathways that independently regulate maturation, behavior, and physiology (Fig. 1).

**Fig. 1.**
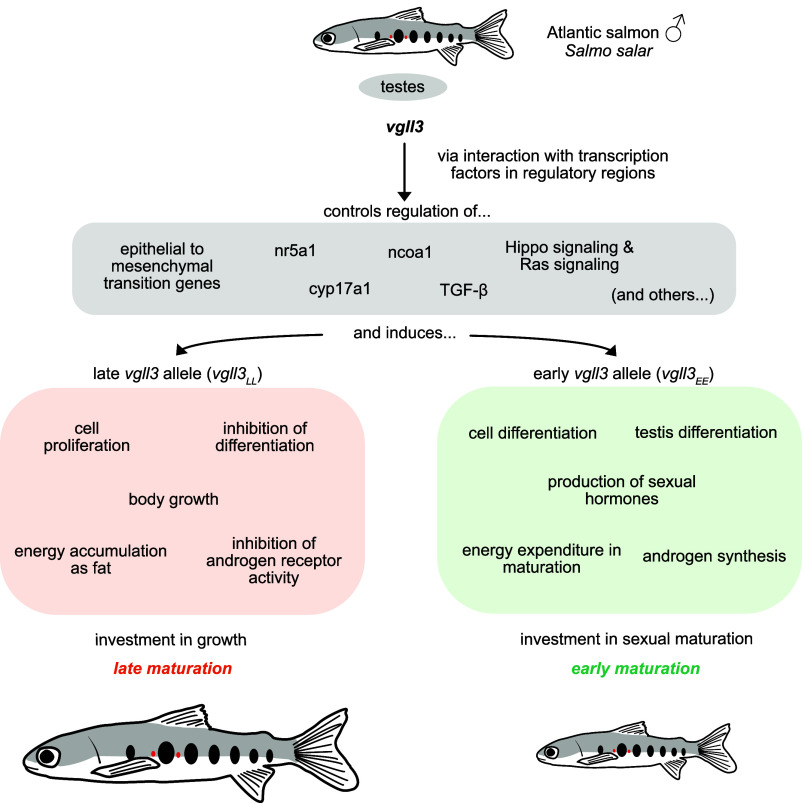
The transcription cofactor *vgll3* is a master regulator of multiple gene pathways that control early and late maturation in Atlantic salmon (*S. salar*). The figure provides a summary of some of the findings by Verta et al. (6).

Life history traits evolve to optimize an organism’s survival and reproductive output in response to natural selection (7). They are complex traits—e.g., size at birth, growth pattern, and age at maturity—that combine variation in physiology, development, and behavior to maximize fitness in a particular environment. Due to their complexity, variation in life history traits is expected to involve multiple genes (polygenic) (5), a genetic architecture that is particularly difficult to study given the statistical challenge of linking the effect of multiple low-effect genes to phenotypic variation. In their study, Verta et al. focus on variation in the age of maturity in Atlantic salmon (*Salmo salar*). The age at which a male Atlantic salmon matures will have a strong influence on reproductive outcome: Maturing late results in larger body size and higher reproductive success, but it also increases the chance of dying before the first reproduction (8). Despite the multiple trait changes that covary with maturation, age of maturation in natural populations of Atlantic salmon is largely explained (40%) by variation at a single gene, the transcription factor (TF) *vestigial-like 3 (vgll3)* (8). While it may be hard to reconcile the large effect of a single gene over such a wide variety of traits, phenotypic variation is often linked to a few genes of large effect across the natural world (5). Being a transcription factor, *vgll3* regulates the expression of other genes. It can thus act as a hub in a regulatory network, integrating multiple upstream signals from various pathways and transmitting them to downstream effector pathways (9).

In PNAS, Verta et al. use state-of-the art genomics to reveal how a single gene determines the life history strategy of salmon through its multifaceted control over molecular pathways that independently regulate maturation, behavior and physiology.

The study of Verta et al. (6) revealed *vgll3*’s master control over multiple regulatory pathways, supporting the notion that it acts indeed as a hub gene. The authors track gene expression changes along the developmental timeline of immature testes in salmon individuals carrying two copies (homozygotes) of *vgll3* variants conferring early (*vgll3_EE_*) or late (*vgll3_LL_*) maturation. EE (i.e., homozygotes for the early allele) individuals show up-regulation of pathways involved in sex hormone production, cell migration, and adiposity, in line with early maturing animals investing fat resources into the production of sex hormones and gonadal maturation, whereas late maturating individuals invest in fat storage and growth. This supports the notion that *vgll3* controls the expression of multiple pathways related with puberty.

But how does *vgll3* control the gene expression of thousands of genes? The authors show that this control happens through direct binding of the VGLL3 protein to enhancer or promoter regions, using ChiPmentation (10). This technique allows mapping the binding sites of TFs or histone markings in the genome, revealing TF binding to promoter and enhancer regions. With this method, the authors were able to confirm that VGLL3 binding to promoter regions altered the gene expression of multiple genes. Furthermore, early-maturing (*vgll3_EE_*) individuals exhibited distinct VGLL3 binding patterns compared to late-maturing (*vgll3_LL_*) individuals, illustrating how functional differences in the VGLL3 protein lead to the coregulation of different functional pathways by the direct control of VGLL3 over gene expression. By analyzing gene expression and binding patterns together, the authors further show that VGLL3_E_ induces stronger up-regulation of sexual maturation pathways than VGLL3_L_. One of the most striking results includes the regulation of the gene *nuclear receptor family 5 group A 1 (nr5a1)*, a transcription factor that controls the production of sex hormones and sexual development. The authors show that the decline in binding of VGLL3 signal over time correlated with decreased expression in *nr5a1* along the developmental timeline of maturation. Similarly, the gene expression pattern and binding patterns of *nuclear receptor coactivator-1 (ncoa1)* were correlated, a gene hypothesized to mediate the balance between investment in fat accumulation in late-maturing individuals and sexual maturation in early-maturing individuals. Finally, the results reveal binding signals of VGLL3 to its own regulatory region, suggesting a self-regulatory mechanism of VGLL3 complementing functional differences between alleles originating from protein-coding mutations.

Using coexpression network analysis of gene expression, an approach that reveals changes in expression across coregulated sets of genes, the authors further illustrate the broad effect of *vgll3* on the expression of entire gene pathways via VGLL3 binding. They reveal, for example, that *vgll3_EE_* individuals show increased expression of the Hippo signaling pathway, which controls the balance between cell proliferation and differentiation. This suggests that *vgll3* directly regulates the timeline of proliferation and differentiation of cells in the testes. Finally, the authors show that the VGLL3 regulatory effect occurs by interaction with other proteins that regulate downstream pathways, revealing its role as a transcriptional cofactor.

Together, this study provides compelling evidence for how a single gene can exert an effect over multiple traits through its role as a transcriptional regulator. It further provides a mechanistic explanation for why certain traits evolve together. Verta et al. show that coevolution of traits may happen due to them being under the control of the same major regulatory proteins. This also contributes to our general knowledge on the evolution of trade-offs in life history strategies. For instance, using the example of the salmon life history cycle from this study, individuals either invest energy in growth or in maturation, but not both. This trade-off may exist simply due to the fact that the regulation of growth and maturation are connected by the central regulatory role of *vgll3*.

Why should a single gene have such a dramatic effect over the developmental trajectory of an entire organism? Previous research (5) has speculated that complex phenotypes may be under the control of pleiotropic gene architectures controlled by master regulators, as the one exemplified in this study. The opposite scenario, involving variation in multiple genes, would require the occurrence of modifications in all the potentially hundreds of different pathways underlying the phenotype. This alternate scenario may be quite unlikely, since it requires the simultaneous occurrence of the right combination of multiple favorable mutations at many different genes and would have a higher chance of generating deleterious fitness effects. On the other hand, the accumulation of regulatory mutations in single transcript cofactors, like *vgll3*, would result in broad modification of the regulation of cellular pathways with very few mutations. In these pleiotropic architectures, natural selection could effectively remove mutations with broad deleterious effects but favor the ones with significance fitness gains. A simple genetic architecture also facilitates the coinheritance of adaptive phenotypes (5, 11).

The findings in this study have broader implications for understanding the genetic basis of complex traits and the evolution of phenotypic diversity. Aspects of genetic architecture of adaptation, such as the number of genes involved, dominance effects, and pleiotropy influence how fast adaptation can occur in response to environmental change (5, 12, 13). Ultimately, dissecting the genetic architecture of ecologically relevant traits such as maturation time allows us to make better predictions of evolutionary outcomes in response to artificial or natural selection (12, 14).

## References

[1] J. Beatty, Replaying life’s tape. J. Philos. **103**, 336–362 (2006).

[2] T. Capblancq, M. C. Fitzpatrick, R. A. Bay, M. Exposito-Alonso, S. R. Keller, Genomic prediction of (Mal)adaptation across current and future climatic landscapes. Annu. Rev. Ecol. Evol. Syst. **51**, 245–269 (2020).

[3] L. Bernatchez, A.-L. Ferchaud, C. S. Berger, C. J. Venney, A. Xuereb, Genomics for monitoring and understanding species responses to global climate change. Nat. Rev. Genet. **25**, 165–183 (2024). 37863940 10.1038/s41576-023-00657-y

[4] L. M. Aubry, C. T. Williams, Vertebrate phenological plasticity: From molecular mechanisms to ecological and evolutionary implications. Integr. Comp. Biol. **62**, 958–971 (2022). 35867980 10.1093/icb/icac121

[5] K. Bomblies, C. L. Peichel, Genetics of adaptation. Proc. Natl. Acad. Sci. U.S.A. **119**, e2122152119 (2022). 35858399 10.1073/pnas.2122152119 PMC9335183

[6] J.-P. Verta , A complex mechanism translating variation of a simple genetic architecture into alternative life histories. Proc. Natl. Acad. Sci. U.S.A. **121**, e2402386121 (2024). 39560647 10.1073/pnas.2402386121 PMC11621623

[7] S. C. Stearns, Life history evolution: Successes, limitations, and prospects. Naturwissenschaften **87**, 476–486 (2000). 11151666 10.1007/s001140050763

[8] N. J. Barson , Sex-dependent dominance at a single locus maintains variation in age at maturity in salmon. Nature **528**, 405–408 (2015). 26536110 10.1038/nature16062

[9] A. Martin, V. Orgogozo, The loci of repeated evolution: A catalog of genetic hotspots of phenotypic variation. Evolution **67**, 1235–1250 (2013). 23617905 10.1111/evo.12081

[10] C. Schmidl, A. F. Rendeiro, N. C. Sheffield, C. Bock, ChIPmentation: Fast, robust, low-input ChIP-seq for histones and transcription factors. Nat. Methods **12**, 963–965 (2015). 26280331 10.1038/nmeth.3542 PMC4589892

[11] S. Yeaman, M. C. Whitlock, The genetic architecture of adaptation under migration-selection balance. Evolution **65**, 1897–1911 (2011). 21729046 10.1111/j.1558-5646.2011.01269.x

[12] M. Kardos, G. Luikart, The genetic architecture of fitness drives population viability during rapid environmental change. Am. Nat. **197**, 511–525 (2021). 33908831 10.1086/713469

[13] M. C. Urban , When and how can we predict adaptive responses to climate change? Evol. Lett. **8**, 172–187 (2023), 10.1093/evlett/qrad038. 38370544 PMC10872164

[14] K. B. Mobley , Maturation in Atlantic salmon (Salmo salar, Salmonidae): A synthesis of ecological, genetic, and molecular processes. Rev. Fish Biol. Fish. **31**, 523–571 (2021).

